# Impact of Growth Conditions on *Pseudomonas fluorescens* Morphology Characterized by Atomic Force Microscopy

**DOI:** 10.3390/ijms23179579

**Published:** 2022-08-24

**Authors:** Houssem Kahli, Laure Béven, Christine Grauby-Heywang, Nesrine Debez, Ibtissem Gammoudi, Fabien Moroté, Hana Sbartai, Touria Cohen-Bouhacina

**Affiliations:** 1 Univ. Bordeaux, CNRS, LOMA, UMR 5798, F-33405 Talence, France; 2 Laboratory of Cellular Toxicology, University of Badji Mokhtar, Annaba 23000, Algeria; 3 Univ. Bordeaux, INRAE, UMR 1332 Biologie du Fruit et Pathologie, 33140 Villenave d’Ornon, France; 4 Laboratory of Biodiversity and Pollution of Ecosystems, University Chadli Bendjedid, El Tarf 36000, Algeria

**Keywords:** Atomic Force Microscopy, *Pseudomonas fluorescens*, Gram-negative bacteria, morphology, bacterial surface, membrane, Lipopolysaccharide, stress

## Abstract

This work is dedicated to the characterization by Atomic Force Microscopy (AFM) of *Pseudomonas fluorescens*, bacteria having high potential in biotechnology. They were first studied first in optimal conditions in terms of culture medium and temperature. AFM revealed a more-or-less elongated morphology with typical dimensions in the micrometer range, and an organization of the outer membrane characterized by the presence of long and randomly distributed ripples, which are likely related to the organization of lipopolysaccharides (LPS). The outer membrane also presents invaginations, some of them showing a reorganization of ripples, which could be the first sign of a bacterial stress response. In a second step, bacteria grown under unfavorable conditions were characterized. The choice of the medium appeared to be more critical in the case of the second generation of cells, the less adapted medium inducing not only changes in the membrane organization but also larger damages in bacteria. An increased growth temperature affected both the usual “swollen” morphology and the organization of the outer membrane. Here also, LPS likely contribute to membrane remodelling, which makes them potential markers to track cell state changes.

## 1. Introduction

*Pseudomonas,* a Gram-negative bacteria adapted to different environments and ecosystems, are, therefore, ubiquitous in many habitats such as soils, sediments, plants and animals as well as fresh and marine waters [1,2]. *Pseudomonas* species can be divided into two groups, depending on whether they are fluorescent or not. Fluorescent species include *P. aeruginosa*, *P. putida* and *P. fluorescens*, which are characterized by the production of high levels of siderophores, such as the water-soluble yellow-green fluorescent pyoverdines.

*P. fluorescens* belongs to the plant-growth-promoting rhizobacteria (PGPR) [3,4]. These bacteria colonize specific rhizospheres, stimulate a plant’s growth and protect it against pathogenic microorganisms [5], improving crop yield [1]. Some strains have also been shown to regulate the population density of the nematode *Pratylenchus penetrans* responsible for root damages [6]. In addition, *P. fluorescens* has a bioremediation potential through the bioaccumulation and the degradation of compounds toxic for plants (metals for instance), by increasing metal absorption by the roots [4,7,8]. Under iron-limiting conditions, pyoverdines also chelate iron very efficiently and are, therefore, essential for proper functioning of bacterial metabolism [9,10]. In addition, this bacterium contributes significantly to the reduction in nitrates and nitrites [11], which are common groundwater pollutants. Another interesting feature relies on *P. fluorescens* capacity to synthesize muporicin, a competitive inhibitor of bacterial isoleucyl-tRNA synthetase. This antibiotic is active mainly against Gram-positive bacteria including methicillin-resistant *Staphylococcus aureus* [12]. All these properties make *P. fluorescens* interesting in the field of biotechnology [13], with multiple applications in food, agriculture, health and environment [14]. At the same time, the status of *P. fluorescens* as a non-pathogenic bacterium can be questioned, with some strains being able to act as pathogens under specific conditions [15] [16].

All these observations underline the urgency of better characterizing *Pseudomonas* species on the genomic, biochemical, physiological and morphological levels. In bacteria, morphology affects critical biological functions, including fitness and stress resistance. Studying bacterial morphology, therefore, appears necessary to better understand how these microorganisms can adapt to different environments. *P. fluorescens* is able to survive in a wide range of experimental conditions, thanks to its large metabolic capacities. The most common growth media used at the laboratory scale are rich and non-selective such as Lysogeny Broth (LB) medium [17,18,19,20] or Mueller–Hinton (MH) medium [21,22]. *P. fluorescens* can also withstand a wide range of temperatures from 4 °C to 42 °C. However, modifying the growth temperature induces variations in the molecular structure of lipopolysaccharides (LPS) of its membrane [23]. An increase in temperature can also modify the attachment properties of *P. fluorescens* to a substrate or a host [24] and, therefore, its capacity to form biofilms or to colonize and invade hosts.

The development of high-resolution imaging techniques including Transmission Electron Microscopy (TEM), Scanning Electron Microscopy (SEM), and Atomic Force Microscopy (AFM) have allowed great progresses in the characterization of the morphology of diverse bacteria, including the *Pseudomonas* species [25,26,27,28,29,30,31,32,33]. AFM presents several advantages compared to the other high-resolution imaging techniques. This is a powerful technique for imaging surfaces in a large range from µm^2^ to nm^2^ with a high spatial resolution, and it also provides access to the mechanical properties of the system at a nanometric scale. Moreover, contrary to TEM and SEM, information obtained by AFM is available in real time and under physiological conditions, explaining its increasing use for the characterization of biological systems [34,35,36,37,38,39]. In the context of the *Pseudomonas* species, AFM imaging studies were often focused on pathogenic species, such as *P. putida* and *P. aeruginosa* [25]. Surprisingly, despite the potential interest for biotechnology and pathogenic capacities, only a few reports concern the characterization of *P. fluorescens* morphology [25,26,27,28,29,30,31,32,33].

The present work aims at characterizing, by AFM, the *P. fluorescens* ATCC 13525 type strain grown on solid medium, under optimal and stressful conditions. Here, AFM will be used as a powerful tool to reveal subtle morphological changes when growth conditions vary in terms of culture medium, temperature and incubation time.

## 2. Results and Discussion

The first part of our work is dedicated to the definition of the best conditions in terms of suspensions used to prepare bacteria deposits. The objective is to obtain, on the same samples, small aggregates and even individual cells with morphological characteristics that can be more easily deduced. In a second step, we present the characterization of *P. fluorescens* after its culture in validated optimal conditions, i.e., in MH medium kept at 28 °C [15,16] for 15 h. This last parameter will be justified in the third part, where AFM was used to reveal the bacterial stress due to less favourable culture conditions.

### 2.1. Determination of the Optimal Bacterial Concentration in Suspensions for AFM

**Figure 1:**
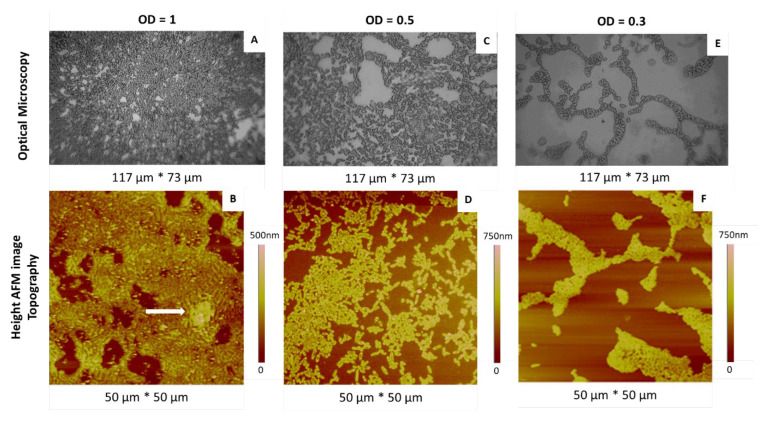
OM (**A**,**C**,**E**) and AFM height (**B**,**D**,**F**) images of *P. fluorescens* deposits made by simple deposition from suspensions at different O.D. values (O.D. = 1.0, images (**A**,**B**); O.D. = 0.5, images (**C**,**D**); O.D. = 0.3, images (**E**,**F**)).

Even if the preparation of bacterial suspensions in water is the last step before imaging, we begin the description of our work by this final step, since the dilution of these suspensions (and, thus, the retained O.D. value) is an essential parameter determining the quality of samples. Indeed, the AFM study cannot be managed using samples made of bacteria multilayers or at a too-low bacteria density, obtained from overly or insufficiently concentrated solutions, respectively. The corresponding solutions were deposited by “simple deposition” on glass slides and bare mica substrates, dried and characterized by OM and AFM. Representative results are shown in Figure 1. As expected, the cell density decreased with decreasing O.D. values. At the highest O.D. value (Figure 1A,B), dense colonies were observed, covering a large part of the surface of the substrate with the presence, locally, of multilayers (white arrow, Figure 1B).

At the intermediate O.D. value (Figure 1C,D), the surface was widely covered, with the coexistence of more or less dense areas and individual bacteria, without a multilayer.

At the lowest O.D. value (Figure 1E,F), bacterial deposits were sparse, with cell aggregates of less than 10 cells as well as many isolated bacteria.

An O.D. value of 0.3 was finally retained, since it enabled us to observe, in a single sample, bacterial populations showing various organizations, from more or less dense areas to isolated cells, with this last configuration being ideal for morphological study.

### 2.2. Characterization by AFM of P. fluorescens Grown under Optimal Conditions

#### 2.2.1. Morphology and Dimensions

**Figure 2:**
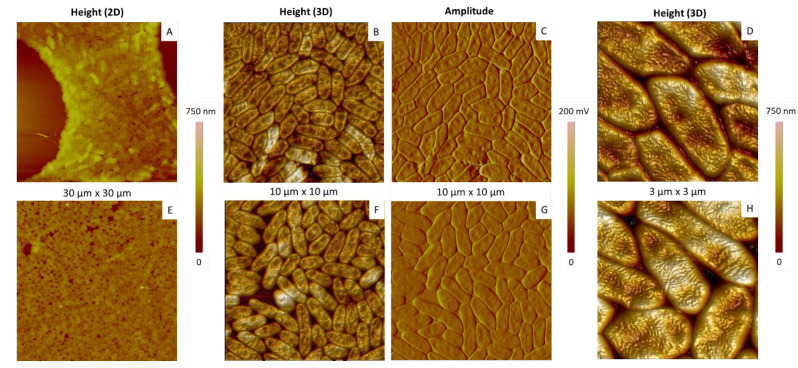
AFM height or amplitude images of *P. fluorescens* deposits obtained by simple deposition (**A**–**D**) or spin-coating (**E**–**H**).

**Figure 3:**
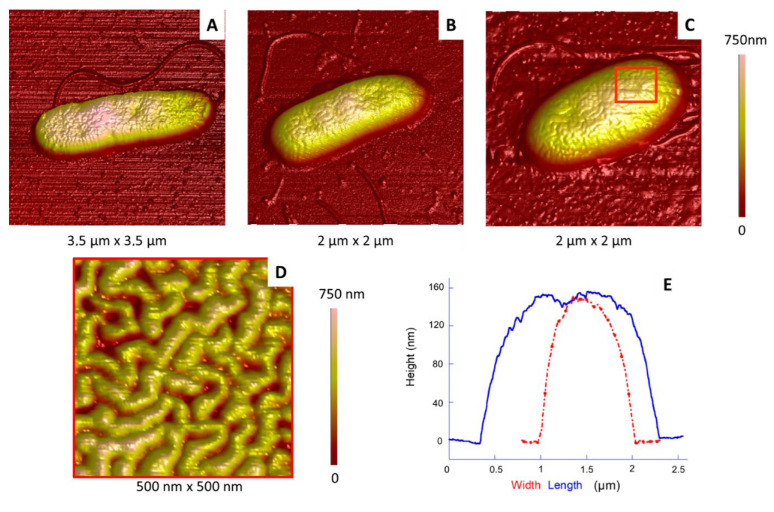
Morphological analysis of *P. fluorescens* cells. (**A**–**C**) AFM 3D topography images of isolated cells; (**D**) AFM phase image corresponding to the red square in (**C**); (**E**) height profiles in length (blue) and width (red) of the cell shown in (**C**).

Representative results are shown in Figure 2 and Figure 3. In the case of a simple deposit (Figure 2, top row), the bacterial population was dense and very compact. Such an organization is relatively frequent in the context of simple deposits favouring aggregation [37]. The rinsing step, performed by aspirating the solvent, could also promote it.

Almost similar results were obtained by spin-coating deposition (Figure 2, bottom row), except that cells were slightly more loosely organized. This lower density is likely due to the fact that most of the solvent is expelled during the rotation of the sample, shifting the bacteria and separating the aggregated cells at the same time.

We then analysed more than 100 aggregated or isolated cells from different samples.

In the case of isolated bacteria, flagella most often remain attached to the cells (Figure 3) and are numerous, as already reported [40,41]. This point is interesting, since *E. coli* bacteria studied using a similar protocol most often appeared to lack their flagella. These appendages are probably lost in the solution during sample preparation and are found subsequently scattered on the surface of the substrate for *E. coli* [39].

The shape of the *P. fluorescens* single cells was rather diverse, depending on their location, whether isolated, at the edges or more in the centre of the aggregates: some of them were elongated and relatively narrow (Figure 3A,B), whereas other ones were more oval (Figure 3C) and rounded (not shown). In the case of aggregated cells, their shape was affected by contacts with their neighbours, leading to more “geometric” shapes because of stronger constraints.

**Figure 4:**
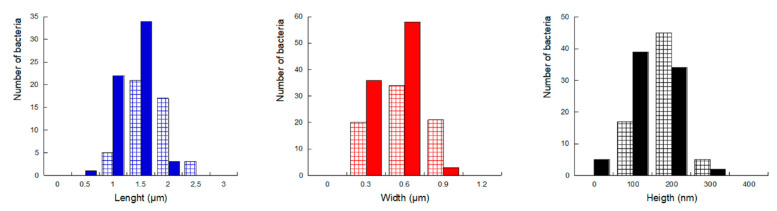
Histograms of *P. fluorescens* length (blue), width (red) and height (black). Measurements were performed on 120 aggregated cells (solid bars) and 40 isolated cells (hatched bars).

Cell width and length could be deduced from the height profiles made along their two axes, as illustrated in Figure 3E. Results are summarized in Figure 4, the typical length and width being around 1.5–2.0 µm and 0.6–0.9 µm, respectively, in agreement with those already reported [42]. Here, the isolated or aggregated organizations also impact the dimensions: aggregated and, consequently, compressed cells are shorter (and/or narrower), with a greater height than individual cells. This height increase is understandable if we assume that the overall cell volume does not change depending on whether cells are aggregated or not [39].

#### 2.2.2. Membrane Organization

An overall observation revealed that the surface of *P. fluorescens* bacteria presented more or less circular invaginations (Figure 2 and Figure 3). These invaginations (from one to five per cell) were observed in the vast majority of bacteria, most often located on the median axis of the bacteria. Based on the height profiles, typical depths are in the range of 5–70 nm, with a diameter around 240 ± 90 nm and centre to centre distances around 500 nm. Such invaginations have been already observed in other Gram-negative bacteria such as *P. putida*, with similarities in terms of number and depth [25], or *Chromobacterium violaceum* [30]. In the latter, invaginations were more numerous (more than 10 per cell), with an average depth around 30 nm.

**Figure 5:**
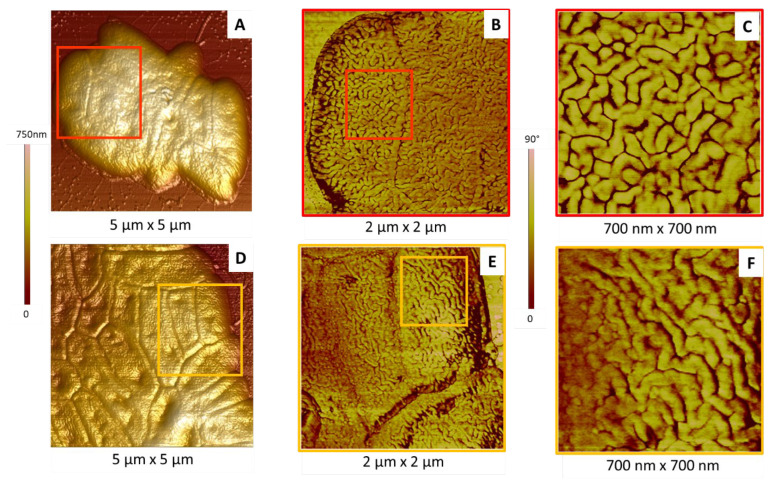
Membrane characteristics of *P. fluorescens* cells. (**A**,**D**) The 3D topographic AFM images of aggregated cells; (**B**,**E**) phase AFM images of areas in the red and orange squares in images (**A**) and (**D**), respectively; (**C**,**F**) phase AFM images of areas in the red and orange squares in images (**B**) and (**E**), respectively.

Another characteristic of *P. fluorescens* is the structuration of the outer membrane surface, showing the presence of “worm-like” undulations made of relatively sinuous tubes separated by grooves (Figure 3D and Figure 5, where tubes and grooves appear as light and dark areas, respectively). The width of these tubes is in the range of 55 ± 6 nm, and the corresponding maximum height is approximately 10 nm. We have already observed this kind of organization leading to a contrast in AFM topography and phase images in a previous study on *E. coli*, another Gram-negative bacteria [39]. Such organization was assigned to the coexistence of two phases with different mechanical properties: undulations, called “ripples”, appearing light in height images, are probably made of well-organized molecules, which make them rather rigid [39,43], whereas the surrounding phase, appearing in dark, is softer or even viscous, made probably of more fluid and/or relatively disorganized molecules [44]. As the outer membrane of these bacteria is made at 75% of LPS, these molecules were proposed to be responsible for this membrane structuration in ripples [39,45,46,47,48].

As illustrated in Figure 5, different organizations of these structures were observed according to the probed cell. Ripples can be more or less long and organized, randomly oriented or aligned (for some of them). In some cases, their length can largely exceed a hundred nanometers (Figure 5E,F) and even approach a micrometer, in particular in the case of aggregated cells (results not shown). These observations underline the inherent heterogeneity of biological samples.

**Figure 6:**
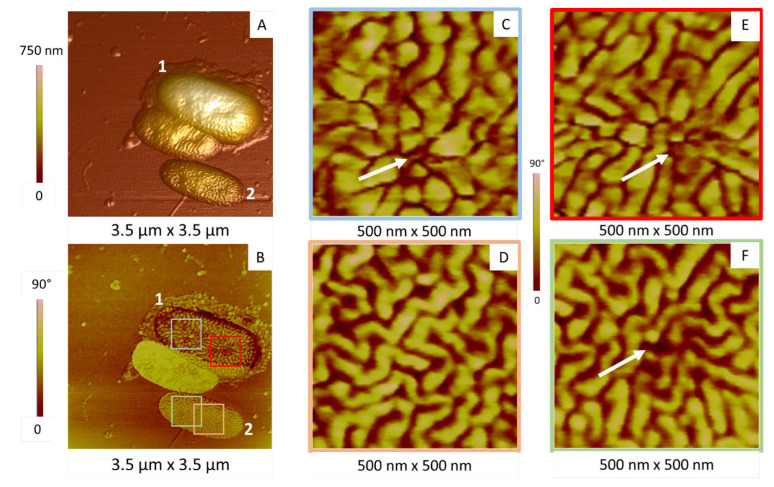
Relationship between membrane organization and invagination, shown by AFM images obtained in air and in tapping mode. (**A**) The 3D topographic images of three bacteria. Characteristics of cells numbered 1 and 2 are described in the main text; (**B**) corresponding phase image; (**C**–**F**) phase images of areas in the red, grey, green and yellow squares in figure (**B**), respectively. White arrows indicate the bottom of the invagination.

This heterogeneity is also visible in Figure 6, where three bacteria are imaged, two of them being numbered 1 and 2. These bacteria share the same overall elongated morphology, even if they have different volumes, with two invaginations at their surface (Figure 6A). However, the corresponding AFM phase image (Figure 6B) shows a clear difference in contrast between cells 1 and 2, the phase of cell 1 being lower than that of cell 2 (appearing in dark and light, respectively). This suggests different membrane mechanical properties in cells 1 and 2, in terms of Young’s modulus and viscoelasticity, with cell 1 being softer than cell 2 [39,43].

Another interesting point is the correlation between the membrane organization and invaginations: in the invaginations observed in cell 1 (Figure 6C,E), the ripples seem to be segmented, shorter and converging to the deepest central zone of the invagination (white arrows). They can also appear as quasi-spherical domains with a diameter in the same range than the width of tubes (around 50 nm) and a height between 4 and 10 nm. Such changes are also observed in one of the two invaginations of cell 2, at a lesser extent (Figure 6F, as compared to Figure 6D).

**Figure 7:**
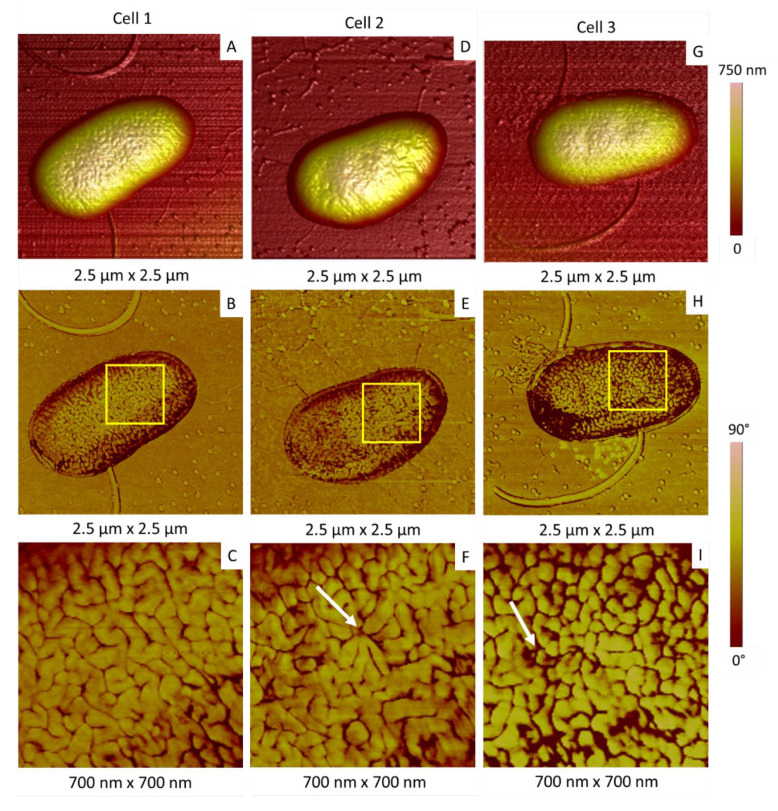
Relationship between membrane organization and invaginations. (**A**,**D**,**G**) AFM 3D topographic images of three bacteria; (**B**,**E**,**F**) corresponding phase images; AFM images shown in (**C**,**F**,**I**) correspond to the areas in the squares indicated in (**B**,**E**,**H**), respectively. White arrows indicate the bottom of the invagination.

This correlation between invaginations and ripples organization has been observed in other samples. Figure 7 shows three typical isolated bacteria having the same morphological characteristics but differing in terms of invaginations. Cell 1 in Figure 7A–C did not show any invagination on its surface, and its membrane is characterized by the presence of “standard” long ripples, previously described on all its surface (Figure 7B,C). Cells 2 (Figure 7D–F) and 3 (Figure 7G–J) presented invaginations on their surfaces, where the organization of membrane is modified with shortened and converging ripples. A last important point has to be mentioned: cell 1 in Figure 6A and cell 3 in Figure 7H were surrounded by material that likely corresponds to excreted vesicles. Such vesicles are the sign of a stressed or not-healthy bacterium [49,50,51].

Our results, in parallel to previous studies, lead us to propose the following hypotheses.

The main part of *P. fluorescens* bacteria observed in our study presents invaginations at their surface, except in rare cases (Figure 7A–C). As previously mentioned, such invaginations have been already observed in other Gram-negative bacteria such as *P. putida* [25] or *Chromobacterium violaceum* [30]. In this last case, the invaginations were assigned to a bacterial stress and considered as a sign of a self-defence procedure induced in bacteria. Following the same idea, Kang and co-workers showed, by SEM and TEM, an irregular morphology and an inhomogeneity on the outer surface of *P. fluorescens* when it is stressed in the presence of lactobionic acid [33].

At the single-cell level, the membrane organization differs according to the observed area. In an area devoid of invagination, ripples are long and randomly oriented. In invaginations, this organization can be maintained or modified with shorter ripples converging towards the centre of the invaginations. We have already observed such a reorganization in the case of *E. coli* bacteria, when they are aged or stressed by their exposure to nanoparticles [39]. These observations suggest that shorter ripples converging in the invaginations could be a sign of a stressed state in *P. fluorescens*, whereas long and randomly oriented ripples and the absence of any invaginations could be a distinctive feature of unstressed bacteria.

When the converging organization of ripples is observed, it is often correlated to the presence of debris or vesicles around the cell. This suggests a process by degrees, where changes in ripples could be the first step of a membrane reorganization leading, finally, to the formation of vesicles.

As in the case of *E. coli*, a different organization of membrane LPS molecules is probably the cause of this membrane restructuring. Such an effect could indeed be related to their capacity to form a more or less extended brush-like structure at the cell surface, with their polymeric behaviour being shown, for instance, by surface-pressure measurements [52].

Finally, a last question remains: if the invaginations are a sign of stressed bacteria, why do they appear when favourable culture conditions were applied? In our case, such an effect could be due to a dehydration induced by the protocol used to image bacteria in the air.

### 2.3. Impact of Less Favourable Culture Conditions on P. fluorescens Morphology

#### 2.3.1. Influence of the Culture Medium

**Figure 8:**
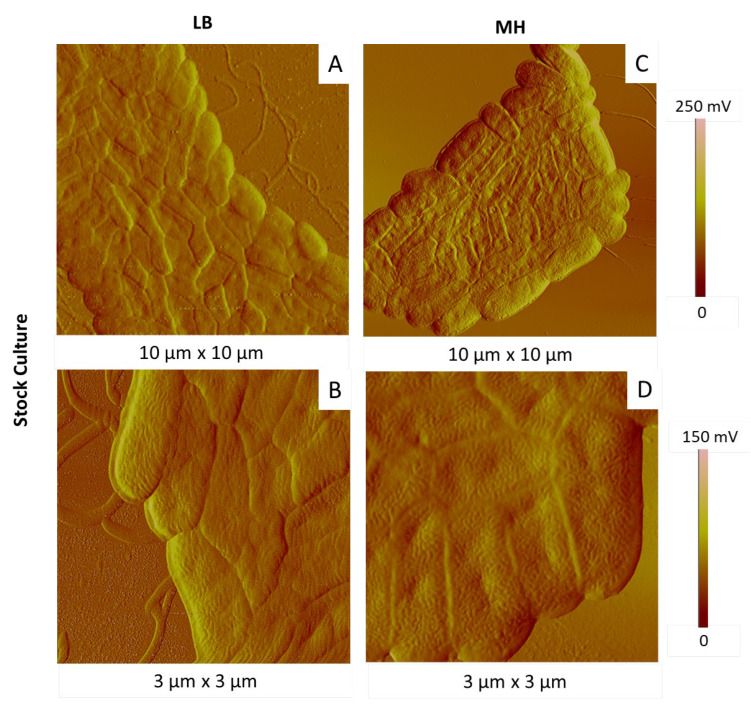
AFM amplitude images of *P. fluorescens* bacteria of first generation deposited by simple deposition from a culture in LB (**A**,**B**) and MH (**C**,**D**) media (15 h, 28 °C).

**Figure 9:**
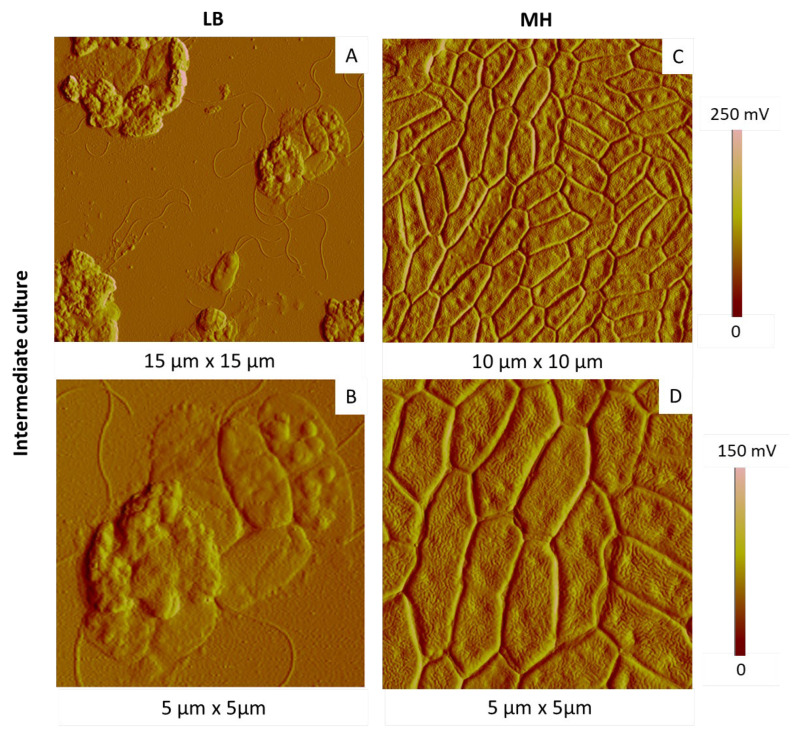
AFM amplitude images of *P. fluorescens* bacteria of second generation deposited by simple deposition from a culture in LB (**A**,**B**) and MH (**C**,**D**) media (15 h, 28 °C).

Keeping culture temperature and time at 28 °C and 15 h, respectively, we then tested two different culture media, LB and MH. We followed two successive generations (with the first generation from stock culture). The most representative AFM images are shown in Figure 8 and Figure 9 for the first and second generations, respectively.

In both cases, first-generation bacteria were organized in homogenous colonies, glued together in a compact arrangement, more or less spread depending on the areas (Figure 8). They had elongated shapes, with rather homogenous dimensions, in the same range as those given previously. A large number of bacteria exhibited invaginations on their membranes, in particular in the case of bacteria grown in MH medium. At this step, it is however difficult to see any real impact of the culture medium on the cell state.

However, a difference appeared with second-generation bacteria: Figure 9A,B (LB medium) revealed a high amount of damaged bacteria, some of them being even collapsed or lysed, their content being spread around “cell ghosts”. The membrane organization was also modified in some cases with the presence of nodules, which were not observed with first-generation bacteria. Figure 9C,D (MH medium) revealed second-generation bacteria that were similar in morphology and dimensions to first-generation ones, organized in relatively dense colonies. Moreover, the structure of the membrane was unchanged as compared to first-generation bacteria.

According to our results, for the first-generation cells, there is no clear difference in terms of cell morphology between the two media. The choice of the medium appears to be more critical in the case of the second-generation cells, with a clear advantage for MH medium, already validated as the most favourable medium for the culture of the *P. fluorescens* strain studied here.

In the two last part of this study (effect of culture temperature and time), we finally kept LB medium for two reasons: we studied only bacteria of first generation, and LB medium could reinforce constraints induced potentially in bacteria by less appropriate culture temperature and time.

#### 2.3.2. Influence of the Culture Temperature

**Figure 10:**
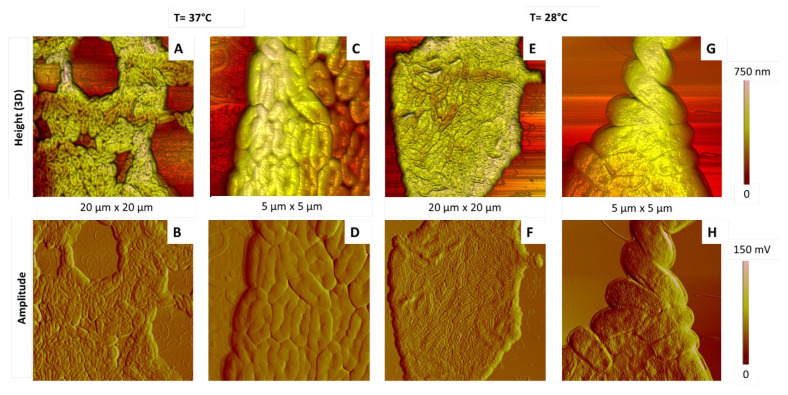
AFM images of *P. fluorescens* bacteria incubated for 15 h in LB medium at 37 °C (**A**–**D**) and 28 °C (**E**–**H**). AFM images are 3D height or topographic ones (**A**,**C**,**E**,**G**) and amplitude ones (**B**,**D**,**F**,**H**).

Figure 10 shows the most representative AFM images of simple deposits after incubation of bacteria at 37 °C and 28 °C. Their comparison shows the following points.

The coverage of the substrate was almost the same for the two kinds of samples. In both cases, the bacterial population was gathered in more-or-less large and dense aggregates (Figure 10A,B,E,F), presenting some gaps or empty areas (Figure 10A,B). As previously, the overall shape of bacteria depended on their location, at the edges or more in the centre of aggregates, with bacteria at the edges suffering weaker constraints.

The average length and width of the bacteria did not seem to be affected by the incubation temperature, with typical values shown in Figure 4. However, cells incubated at higher temperature were deflated (Figure 10A–D), as compared to bacteria incubated at 28 °C (Figure 10E–H), and some of them were even lysed, spreading their inner content around them (Figure S1). This observation was confirmed by height profiles showing that bacteria are twice as thick at 28 °C (140–180 nm) than at 37 °C (60–100 nm) (Figure S2).

As illustrated by the comparison of Figure 10D,H, the cell outer membrane of bacteria incubated at 37 °C was relatively smooth, while it exhibited a more heterogeneous structure after incubation at 28 °C.

In summary, bacteria were deflated as compared to the usual “swollen” morphology, when grown at 37 °C. The organization of the outer membrane is also affected, being smoother. These changes could be an effect of the growth temperature, since this temperature influences the composition of LPS and, thus, their physicochemical properties. Finally, a high culture temperature is clearly a cause of stress for *P. fluorescens* bacteria, in agreement with previous studies showing an optimal growth of *P. fluorescens* at 28 °C and a decreased cellular growth at higher temperature, which finally stopped at 42 °C [53,54,55].

#### 2.3.3. Influence of the Growth Incubation Time

**Figure 11:**
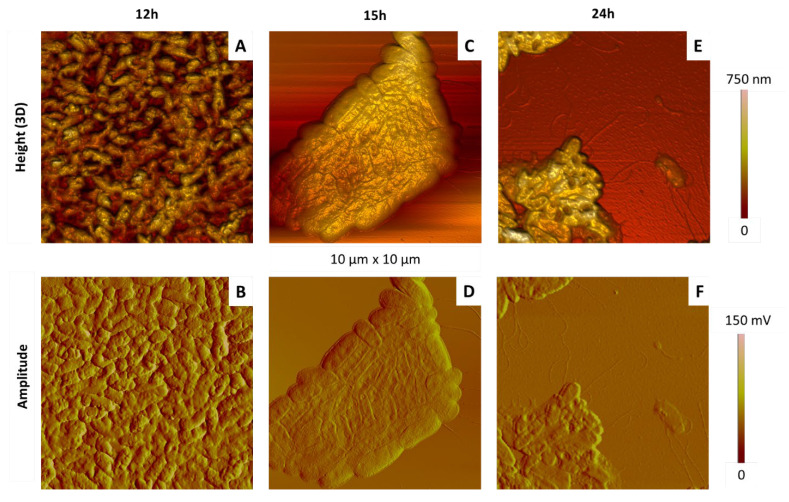
Effect of the incubation time on the morphology of aggregated *P. fluorescens* bacteria deposited by simple deposition. The bacteria were cultured in LB medium at 28 °C during different culture times: 12 h (**A**,**B**), 15h (**C**,**D**) and 24 h (**E**,**F**). AFM images are 3D height or topographic ones (**A**,**C**,**E**) and amplitude ones (**B**,**D**,**F**).

**Figure 12:**
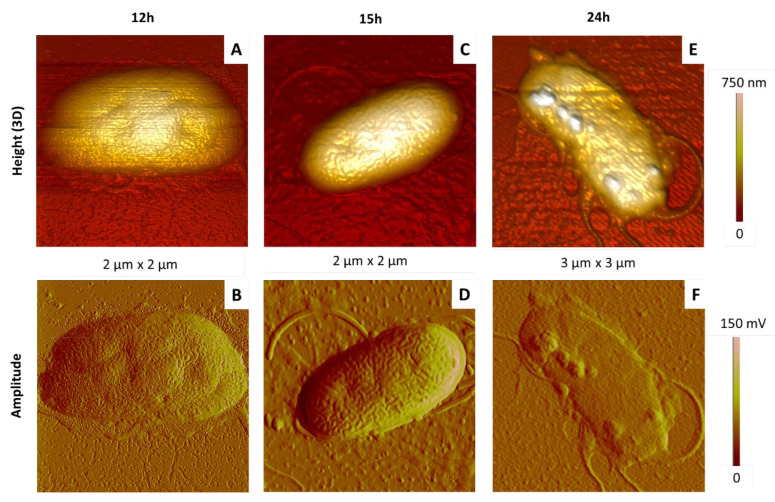
Effect of the culture time on the morphology of isolated *P. fluorescens* bacteria deposited by simple deposition. The bacteria were cultured in LB medium at 28 °C during different incubation times: 12 h (**A**,**B**), 15 h (**C**,**D**) and 24 h (**E**,**F**). AFM images are 3D height or topographic ones (**A**,**C**,**E**) and amplitude ones (**B**,**D**,**F**).

Figure 11 and Figure 12 show typical AFM images of bacteria incubated during 12, 15 and 24 h at 28 °C.

Their comparison shows the following points. In the case of samples obtained after the shortest incubation time (12 h), bacterial colonies were quite dense and compact (Figure 11A,B). Bacteria were heterogeneous in size and shape (elongated or rather round), and some of them appeared sagged, presenting deeper invaginations than those previously described (Figure 12A,B) or even nodules at their rough surface.

At the intermediate time (15 h), AFM images of aggregated (Figure 11C,D) and isolated (Figure 12C,D) cells were typical from healthy bacteria, with the usual dimensions and shape, and the heterogeneous membrane structure mentioned previously.

After 24 h, bacteria colonies are clearly impacted (Figure 11E,F): cells in close contact with others were collapsed and even emptied of their cellular content, some of them being lysed. Similar observations can be made in the case of individual bacteria (Figure 12E,F), showing for instance that the cellular height is divided by four between samples shown in Figure 12C–F. We also note the presence of nodules on the bacterial surface, with typical diameter and height around 200 nm and 50 nm, respectively.

Our results, consistent with those obtained in other works [53,54,55], show that a growth time of 15 h on solid medium is optimal to observe bacteria morphology typical from healthy cells. This justifies, finally, the time condition used in Section 3.2.

## 3. Materials and Methods

### 3.1. Bacterial Strain, Reagents and Growth Conditions

*P. fluorescens* (ATCC 13,525) was kept aerobically at −80 °C in Mueller–Hinton (MH) nutritive broth (Sigma Aldrich; Saint-Quentin-Fallavier, France), reference 70,192, containing beef infusion solids 2.0 g/L, starch 1.5 g/L and casein hydrolysate 17.5 g/L, at a final pH of 7.4 ± 0.2, and 25% glycerol. Stock solutions were used to inoculate either a LB liquid medium (tryptone 10 g, yeast extract 5 g, sodium chloride 10 g, pH 7.2 at 25 ° C) or a MH medium, all purchased from Sigma Aldrich (France), as a ready-to-use powder reconstituted using ultrapure water (Milli-Q water, pH 5.5, resistivity > 18.2 MΩ.cm). Overnight liquid cultures (28 °C under shaking at 180 rpm) were then spread at the surface of the same medium containing 15 g/L of agar (Sigma Aldrich, France) in Petri dishes, for an incubation at 28 or 37 °C, during 12, 15 or 24 h. These cells will be called first generation hereafter. In some experiments, we also studied the second generation (corresponding to a second culture of bacteria from Petri dishes under the same conditions).

Bacteria were then stripped off the agar surface and, finally, suspended in ultrapure water. These suspensions were diluted at a concentration equivalent to an optical density (O.D.) of 1.0, 0.5 and 0.3, measured at a wavelength of 600 nm with a UV3600 Shimadzu spectrophotometer. An O.D. of 1.0 is equivalent to a bacterial concentration of 1.2 × 10^9^ CFU/mL [56,57].

### 3.2. Preparation of Samples for Imaging Experiments

Suspensions in ultrapure water were then deposited on different substrates depending on the imaging method. Nude mica (Electron Microscopy Sciences, France) was used for AFM experiments, as freshly cleaved mica presents a highly hydrophilic, perfectly clean and homogeneous surface with a low average roughness of 0.3 nm/m^2^, suitable for AFM. Glass slides (Thermo Scientific, France) were used as substrates for optical microscopy (OM).

In each case, 5 µL droplets of the bacterial solution were deposited using two methods: a simple deposition or a deposition assisted by spin-coating at a speed of 200 rpm for 45 s.

Weakly attached bacteria were removed by a gentle rinsing with ultra-pure water, and substrates were then dried in a desiccator for 2 h before imaging in air. Four independent samples were prepared and analysed systematically.

### 3.3. Optical Microscopy (OM)

We used a fixed stage vertical microscope (BX51WI from Olympus) equipped with a 100W mercury lamp (U-LH100HG), a BX-RFA illuminator and two LMPF1 objectives: ×50 (image of 234 µm × 146 µm) and ×100 (image of 117 µm × 73 µm). The microscope was coupled to a high-resolution ORCA-Flash 2.8 camera equipped with the HC Image Live V3.0 software (Hamamatsu Photonics, Massy, France).

Several areas of each sample were systematically observed to ensure their homogeneity. This technique was used for all samples to verify their quality before AFM experiments.

### 3.4. Atomic Force Microscopy 

AFM experiments were performed using a Bioscope II device mounted on an Olympus IX71 inverted optical microscope operating with the NanoScope V controller (Veeco-Brucker, Santa Barbara, CA, USA). Displacement of the samples was ensured by a piezo scanner (maximum XYZ scanning range of 150 µm × 150 µm × 12 µm), AFM measurements being carried out in air, in tapping mode using commercial silicon cantilevers (NCLV, Bruker), with a stiffness of 48 N/m and at a working frequency of 178 kHz. The scanning speed was between 0.5 and 1.0 Hz, and the resolution of the collected images was 512 × 512 pixels.

Several areas of the samples were systematically observed, first in a large scan (maximum dimensions of 150 µm × 150 µm), then reduced to the area of interest (minimal dimensions of 500 nm × 500 nm). Several signals were collected at the same time and resulting (and complementary) images are shown below.

Height images were obtained at a relatively high oscillating amplitude of the cantilever, with the contrast corresponding to the topography of the sample [39,43,58].

Phase images were built from the phase difference between the piezo excitation signal (probe’s vibration) and that of the cantilever response, when the tip is in contact with the sample surface. Consequently, the contrast in phase images is related to changes in the nanomechanical properties of the surface of the sample.

Amplitude images were built from the error signal, due to the difference between the oscillation amplitude signal of the cantilever and that of the set point value via the regulation loop. Such images most often provide information complementing height images by highlighting all the topographic variations (small or large) and giving a three-dimensional effect.

## 4. Conclusions

This work was dedicated to the comparative characterization, by Atomic Force Microscopy, of *P. fluorescens*, grown under optimal and stressful conditions. These bacteria having a high potential in biotechnologies, so this study can help to better understand the processes leading to their adaptation to changing environments. Under optimal growth conditions, AFM revealed a more-or-less elongated morphology, with typical dimensions in the micrometer range, and a particular organization of the outer membrane characterized by the presence of long and randomly organized ripples, as also shown in other Gram-negative bacteria presenting LPS at their surface. The outer membrane presents also invaginations, some of them being characterized by a reorganization of ripples, which could be the first sign of a stress in bacteria, as precursors of excreted vesicles. A culture in stressful conditions impacts bacteria at different levels, inducing a high number of damaged bacteria and changes in the membrane organization.

Finally, this work also confirms AFM as a powerful tool to reveal subtle changes, at the nanometer scale, at the surface of bacteria.

As a possible extension of this work, AFM could be used to probe the effect of heavy metals on this species, which is known in particular for its soil bioremediation potential. It would be also useful to extend this study to biofilms of *P. fluorescens*, with a relatively dense organization that is also known to be more resistant to stressful environmental conditions.

## Data Availability

Not applicable here.

## Supplementary Materials

The following supporting information can be downloaded at: https://www.mdpi.com/article/10.3390/ijms23179579/s1.
