# Supplementary material for: Impact of Growth Conditions on Pseudomonas fluorescens Morphology Characterized by Atomic Force Microscopy
Source: Int J Mol Sci. 2022 Aug 24;23(17):9579. doi: 10.3390/ijms23179579 (PMC9455637; doi:10.3390/ijms23179579)
Supplement: Supplementary file 1 [file ijms-23-09579-s001.zip › ijms-1867750-supplementary.pdf]

SUPPLEMENTARY MATERIALS

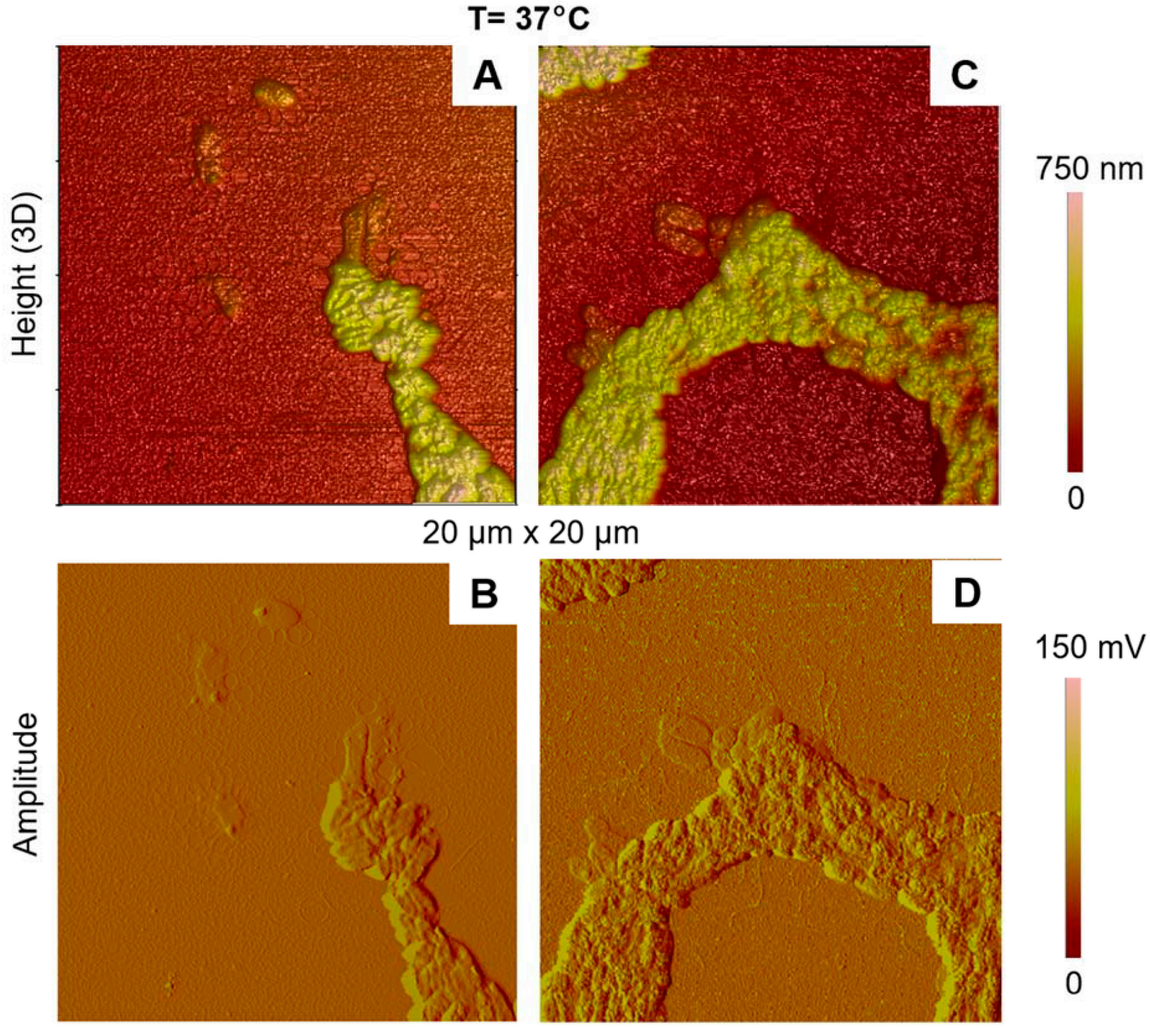

Figure S1. AFM images of *P. fluorescens* bacteria incubated for 15 hours in LB medium at 37 °C. AFM images are 3D height or topographic ones (A, C) and amplitude ones (B, D).

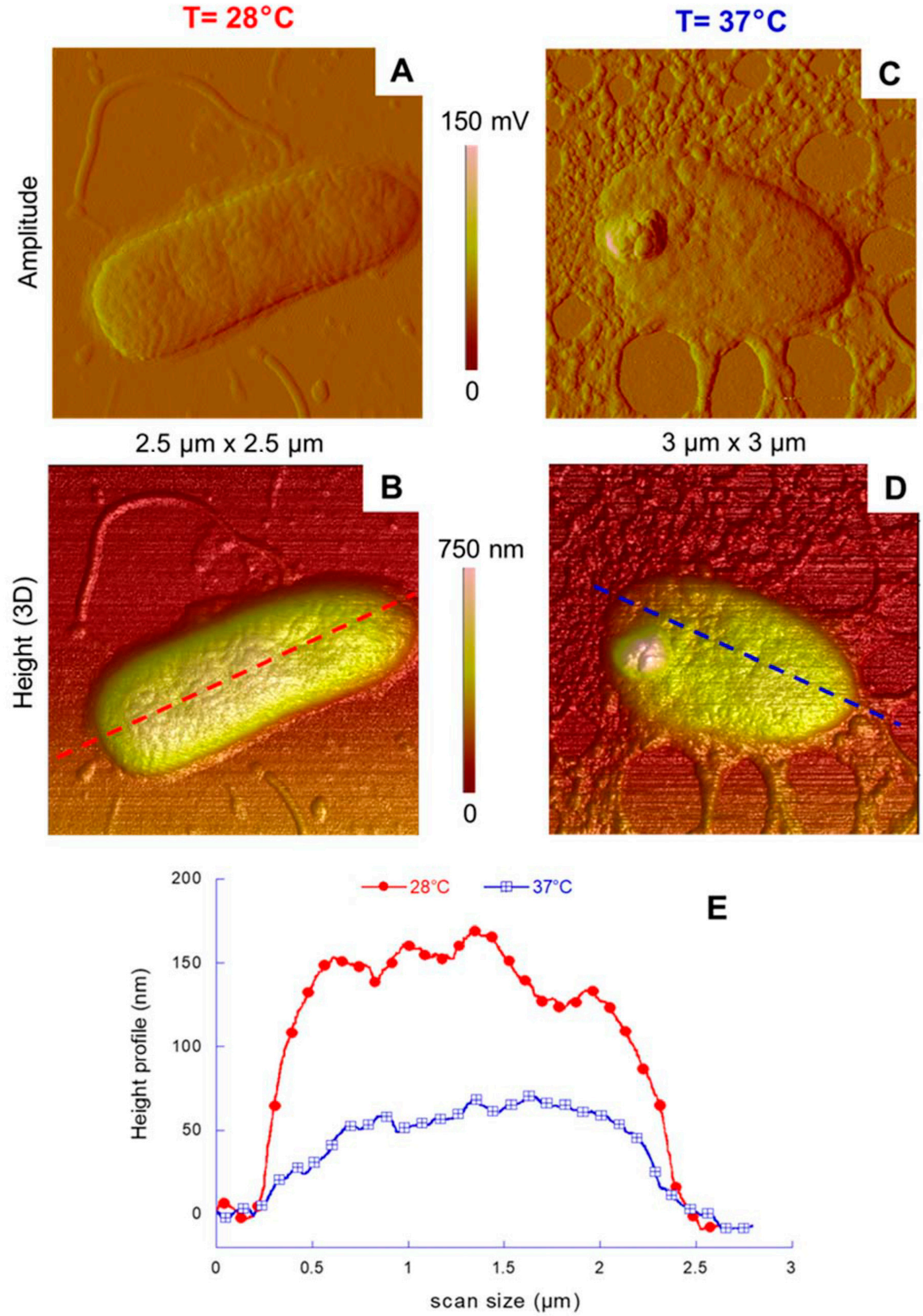

Figure S2. AFM images of *P. fluorescens* bacteria incubated for 15 hours in LB medium at 28 °C (A, B) and 37 °C (C,D) and. AFM images are amplitude ones (A, C) and 3D height or topographic ones (B, D). (E) Height profiles of the two bacteria imaged in (B) and (D).
